# Human Immunodeficiency Virus and Uveitis

**DOI:** 10.3390/v15020444

**Published:** 2023-02-05

**Authors:** Mingming Yang, Koju Kamoi, Yuan Zong, Jing Zhang, Kyoko Ohno-Matsui

**Affiliations:** Department of Ophthalmology & Visual Science, Graduate School of Medical and Dental Sciences, Tokyo Medical and Dental University, Tokyo 113-8510, Japan

**Keywords:** uveitis, human immunodeficiency virus, HIV-related uveitis, opportunistic infections

## Abstract

Uveitis is one of the most common ocular complications in people living with the Human immunodeficiency virus (HIV) and can be classified into HIV-induced uveitis, co-infection related uveitis, immune recovery uveitis, and drug-induced uveitis. The introduction of antiretroviral therapy has considerably changed the incidence, diagnosis, and treatment of different types of HIV-related uveitis. Furthermore, the specific immune condition of patients infected with HIV makes diagnosing HIV-related uveitis difficult. Recent studies have focused on the growing prevalence of syphilis/tuberculosis co-infection in uveitis. Simultaneously, more studies have demonstrated that HIV can directly contribute to the incidence of uveitis. However, the detailed mechanism has not been studied. Immune recovery uveitis is diagnosed by exclusion, and recent studies have addressed the role of biomarkers in its diagnosis. This review highlights recent updates on HIV-related uveitis. Furthermore, it aims to draw the attention of infectious disease physicians and ophthalmologists to the ocular health of patients infected with HIV.

## 1. Introduction

Human immunodeficiency virus (HIV) infection is a retrovirus-induced multisystemic disease. In 1981, the first HIV case was identified in Los Angeles, and HIV had infected more than 38 million people by 2019 [1]. At the peak of the pandemic, HIV resulted in the death of >1.7 million people in 2005 [2]. Over the past two decades, the incidence of HIV infection worldwide has stabilized due to the introduction of antiretroviral therapy (ART). However, in developing countries, especially those in sub-Saharan Africa, HIV infection is still a great burden. HIV prevalence in sub-Saharan Africa is approximately 5%, which is much greater than that in other regions [3]. Moreover, in 2019, less than half of the people living with HIV (PLWH) had suppressed viral loads in >28% of countries, indicating that they were at risk of transmitting the virus [3].

The survival time of patients with HIV is increasing with the application of ART. However, their vision quality has become a matter of concern. Almost all patients with HIV complain of ocular manifestations that cause trouble in their daily life [4,5]. Uveitis is the main cause of blindness and is a common ocular complication of HIV [4,6]. The outcomes of HIV-associated uveitis are not ideal. Hence, ophthalmologists should be aware of this disease, improve patients’ awareness, and achieve the best management to prevent vision loss. This paper focuses on the latest developments in the different causes of uveitis in patients with HIV.

## 2. Prevalence of HIV and Its Related Ocular Compilations

More than half of HIV-infected patients present with ocular manifestations. Although this number has rapidly decreased since the introduction of ART, it remains a serious problem for patients with HIV. Ocular complications of patients with HIV can occur at any stage and segment in the eye [7], involving the eyelids, eyelashes, and anterior and posterior eye segments. The prevalence of different ocular manifestations varies in different regions, with the most common presentations including dry eyes, cytomegalovirus (CMV) retinitis, uveitis, and keratitis [4,7,8]. CMV retinitis is an acquired immunodeficiency syndrome (AIDS)-defining opportunistic infection. In the pre-ART era, CMV retinitis was the most common ocular manifestation of HIV infection, with a prevalence of 30% [9]. However, with the emergence of ART, the burden of CMV retinitis has decreased by >95% [10], although it is still one of the most common ocular manifestations in PLWH [4,11,12,13]. Actually, the involvement of the anterior segment is more common than that of the posterior segment in the post-ART era. According to a study from Tunisia, dry eye (8%) is the most common manifestation in the anterior segment [14]. The prevalence of uveitis in patients with HIV is 1.1–24% [4,7,14]. However, considering that two-thirds of PLWH live in HIV high-burden countries [3], where the awareness of uveitis still needs to be improved, it is still an emergency for ophthalmologists to have more access to patients with HIV-related uveitis. 

Compared with HIV-negative patients, HIV-positive patients have a five-to-six-fold higher chance of being diagnosed with uveitis [15]. It has been well demonstrated that HIV could enter the central nervous system (CNS) and replicate within ocular tissue [16,17]. Meanwhile, the depletion of CD4+ T cells induced by HIV and consequent opportunistic infections and co-infections have increased the prevalence of uveitis as an HIV-related ocular complication [18]. However, patients may only complain of decreased vision at the onset of uveitis; thus, it is easily neglected by the clinician, leading to blindness. The causes of uveitis in HIV infection can be divided into four main categories: HIV-induced uveitis, co-infections-related uveitis, immune recovery uveitis (IRU), and drug-induced uveitis.

## 3. Causes of Uveitis and Its Clinical Manifestation

### 3.1. HIV-Induced Uveitis

It has been demonstrated that HIV can break the blood-brain barrier (BBB) and consequently cause nerve degeneration. As early as 1988, detectable HIV viral loads were found in ocular tissues [19]. HIV was found in the vitreous, retina, iris, conjunctiva, and even tears [20,21,22], indicating that the virus could cross the blood-retina barrier and enter the intraocular space. Several recent studies have focused on the intraocular replication of HIV. Rothova et al. found that the intraocular HIV-1 RNA viral load largely exceeds the plasma HIV-1 RNA load in HIV patients with uveitis and no evidence of other antigens causing uveitis [17]. A research from 2012 has demonstrated the clinical manifestations of HIV-induced uveitis. All six patients had mild anterior uveitis, and none received ART. Inflammation is sensitive to ART rather than traditional steroid treatment [23]. According to research from Thailand, intraocular HIV viral load was detectable in 32% of uveitis patients with HIV [22]. It is worth noting that four of them had higher intraocular HIV viral loads than plasma, and intraocular HIV viral loads rapidly decreased after ART, suggesting that uveitis was caused directly by HIV. Plasma viral load and breakdown of the BBB are suggested to be positive factors of intraocular viral load. However, they did not find a correlation between CD4+ T cells and intraocular viral loads. Furthermore, this implies that HIV can replicate within the ocular space and cause uveitis. Overall, when patients with HIV who have not received ART show uveitis manifestation, especially anterior uveitis, or uveitis without retinal manifestation, HIV-induced uveitis should be suspected.

The HIV Tat protein, a transactivator protein, is important for HIV replication and infection. Research has confirmed that the HIV Tat protein participates in the breakdown of the blood-retina barrier [24]. In addition, within the ocular environment, HIV Tat induces the death of retinal microvascular endothelial cells via N-methyl-D-aspartate receptors, activates Muller cells, and increases pro-inflammatory cytokines [25]. However, further investigations of the detailed mechanism are required.

### 3.2. Co-Infection-Related Uveitis

#### 3.2.1. Syphilitic Uveitis

Syphilis incidence has been reported to increase since the beginning of the 20th century in both developed and developing countries [26], especially among males who have sex with males [27]. Over the last two decades, this increased incidence was primarily due to high-risk sexual behavior [28]. PLWH have a 77 times higher chance of being co-infected with syphilis than HIV-negative individuals, according to a report from the United States [29]. Syphilitic uveitis is one of the most common ocular complications of syphilis; as many as 9% of patients with HIV/syphilis co-infection have ocular manifestation [30,31], while according to a study from The Netherlands, 58.8% of patients with syphilis complain of blurred vision that affects driving [32]. Considering the high prevalence of HIV in areas with a high prevalence of syphilis infection, the number of HIV/syphilis co-infected patients is sizable. Hence, syphilitic uveitis in patients with HIV deserves more attention.

Syphilis serologic testing involves treponemal and nontreponemal tests. Treponemal tests detect infection by testing immunoglobulin (Ig)M and IgG directly against *T. pallidum*, while nontreponemal tests are indirect tests that measure biomarkers released from cells damaged by *T. pallidum*. Although treponemal tests are more sensitive than nontreponemal tests during early infection, they are more complicated [33]. Serologic diagnosis of syphilis requires two positive tests, at least one of which must be a treponemal test. Typically, serological testing for syphilis is based on initial screening with a non-treponemal test and confirmation with a treponemal test if the screening result is positive. In recent two decades, reserve sequence screening, a newer treponemal test, has been used in some laboratories. Reserve sequence screening is based on the application of automatable treponemal enzyme and chemiluminescence immunoassays (EIA/CIA). Considering its advantages of simple operation and lower price, reserve sequence screening is typically performed as the first test. Reserve sequence screening is more sensitive in the early or latent stage [34]. However, compared with non-treponemal tests, the reserve sequence screening algorithm has a greater potential for false positive results [35]. Therefore, the Centers for Disease Control and Prevention recommends that a specimen with a positive result in reverse sequence screening should be tested again with a quantitative nontreponemal test [34,36,37].

**Figure 1:**
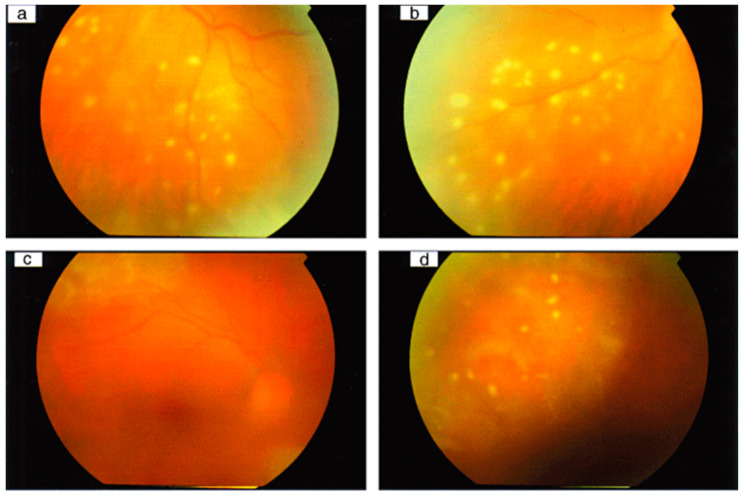
Syphilitic panuveitis patients with punctuate inner retinitis. Color fundus photographs of the left eye of a patient with syphilis, showing (**a**) punctuate inner retinitis overlying deeper retinal inflammation inferotemporally and (**b**) inferonasally without deeper involvement. Color fundus photographs of the right eye of another patient with syphilis, showing (**c**) posterior pole and (**d**) superior views with inner punctuate retinitis and deep diffuse retinitis superior to the superotemporal arcade, with ARTerial sheathing. (Adapted with permission from Ref. [27]. 2010, Edward H Hughes et al.).

As the name of “the great imitator” indicates, the clinical findings of syphilitic uveitis are highly diverse and nonspecific. Any segment or disease stage may be associated with syphilitic uveitis. Thus, diagnosing syphilitic uveitis is difficult, and it is easily overlooked and misdiagnosed. The involvement of panuveitis and posterior uveitis is the most common manifestation of syphilitic uveitis (Figure 1). In contrast, anterior uveitis is more common among HIV-negative patients with syphilis [27]. Chorioretinitis and bilateral involvement are also more common in syphilitic uveitis than in general uveitis [27,32,38,39,40]. Serological testing is useful for diagnosing syphilitic uveitis, while patients with HIV might need more time to respond to the test [41]. Co-infection with HIV and syphilis is common. Hence, routine testing for either virus is highly recommended for patients diagnosed with HIV or syphilis. 

The majority of uveitis clinicians choose antibiotic treatments for their patients. The most commonly used antibiotic is intravenous penicillin, continued for 10–14 days or longer depending on the patient’s status [27,42]. Administering ART does not seem to prevent syphilis infection; however, when combined with intravenous penicillin, ART could help relieve early manifestations [43,44]. Steroids were administered to selected patients. However, a study showed that administering steroids as local injections may even worsen the final outcomes, although this may be related to the time frame between antibiotic treatment and steroids [45]. Syphilitic uveitis is often accompanied by syphilitic retinitis and ocular neurosyphilis, which are risk factors for adverse outcomes [25]. Better baseline best-corrected distance visual acuity (BCVA) and visual improvement in the early stages were significantly correlated with better outcomes. Therefore, early diagnosis and treatment are vital for the outcomes [46].

#### 3.2.2. Tubercular Uveitis (TBU)

HIV spread in Africa began in the 1970s. Currently, about two-thirds of patients with HIV live in Sub-Saharan Africa [47], which is also an area with a high prevalence of tuberculosis (TB). HIV infection is an independent risk factor for active TB [48], and from the World Health Organization (WHO) report in 2013, *M. tuberculosis* co-infection was the leading cause of death in patients with HIV. Meanwhile, compared with immunocompetent people, patients with HIV, especially those whose CD4+ T cell count is <200 cells/µL, have a two to three times higher chance of getting TBU with more rapid progress [49,50]. By 2010, approximately 14 million individuals had HIV and TB co-infection [50]. In fact, due to the absence of authoritative uniform guidelines for diagnosing TBU and false-negative results caused by the low CD4+ T cell count, the number of HIV and TB co-infection cases might be underestimated [51]. 

**Figure 2:**
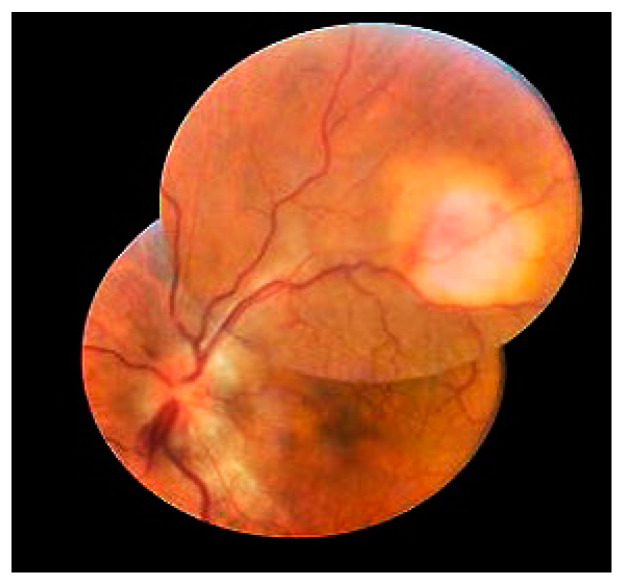
Fundus photograph of the left eye at presentation showing a chorioretinal granuloma, papilledema, retinal vasculitis, and minimal vitritis. (Adapted with permission from Ref [64]. 2014, Erik Schaftenaar et al.).

The detection of *M. tuberculosis* from aqueous/vitreous sampling or detection of the *M. tuberculosis* gene (MPB64 or IS6110) with PCR testing is the most affirmative evidence for diagnosing TBU. However, it is difficult to confirm microbiologic evidence from such limited materials. Furthermore, most studies’ diagnostic criteria for TBU included ocular manifestations suggestive of uveitis, tuberculosis-related systematic manifestation (caught, fever, night sweats, and weight loss), and laboratory investigations suggesting TB infection [52,53,54]. The prevalence of posterior segment involvement and panuveitis in TBU was higher than that in general uveitis (Figure 2). Compared with non-TBU uveitis, chronic uveitis is also more common in patients with TBU. 

Commonly used laboratory investigation methods include the tuberculin skin test (TST) and interferon-gamma release assay (IGRA). However, the sensitivity and specificity of the TST are not satisfactory for diagnosing TBU. According to previous reports, the general sensitivity of the TST is between 7.59–87.0% [52,54]. However, this number drops to 17.2% in PLWH. Hence, a positive TST result in a PLWH could only suggest that this patient has a higher risk of developing TB and does not necessarily indicate active TB disease, especially for those with CD4+ T cell counts <200 cells/mm^3^ [55,56]. IGRA may be the best available laboratory investigation for TB since its sensitivity ranges from 65% to 93%, and its specificity is up to 99% [57,58]. However, IGRA is still unideal for patients with HIV. The presence of HIV reduces the sensitivity of IGRA to 63% [59]. Mainly, this is because IGRA depends on the immune response to the *M. tuberculosis* antigen, and the specificity of this test is decreased due to cross-reactivity with other co-infection viruses. According to La Distia Nora et al., compared with the idiopathic uveitis group, the TBU group had less positive QFT-G results, and the positive result was more related to previous or latent infection of *M. tuberculosis* instead of active TB lesions [53]. Other systemic investigations, such as chest computed tomography scans, have also provided important tips for diagnosing TBU.

Antituberculosis Treatment (ATT) with or without steroids is highly recommended for the treatment of TBU. A study in 2018 showed that ATT treatment was beneficial, even for HIV-infected patients and latent TB [60,61]. Experts recommend that the decision to start ATT should depend on the phenotypes of TBU and the incidence of TB in the patients’ geographical region [62]. Steroids could be administered as an adjunct therapy, especially for patients with signs of an autoimmune reaction to the molecular mimicry of latent TB [52]. Prophylactic treatment with isoniazid is recommended for patients with HIV regardless of whether the TST result is positive for potential lifesaving benefits [56]. Considering the interaction between ART and Rifampicin, clinicians should assess whether ART is needed before ATT [63].

### 3.3. Immune Recovery Uveitis

Although it has reduced the prevalence of opportunistic infections, ART has also increased the incidence of immune reconstitution inflammatory syndrome (IRIS). The prevalence of IRIS from global research is unavailable, and the number based on a single study is significantly different. Approximately 10–32% of patients with AIDS who received ART experienced IRIS [65,66]. IRU is one of the most common ocular manifestations of IRIS. It is defined as any new inflammation in an eye with controlled CMV retinitis or other ocular infections, not attributable to an alternative cause, following substantial recovery of immunity [12]. Currently, IRU has become an important cause of vision loss in patients with HIV who received ART [67].

The pathology of IRU is not fully understood, but studies have suggested that it is associated with recovering the immune response to CMV infection. Destruction of existing CD4+ T cells and the inability to activate new CD4+ T cells caused by HIV infection reduced the absolute CD4+ T cell count and increased the risk of opportunistic infection [18]. CMV, the most common opportunistic infection, causes a break in the blood-retina barrier and intraocular inflammation [68]. Treatment with ART effectively inhibited HIV infection and increased CD4+ T cell counts. With the recovery of immune function, the immune system can respond to existing antigens such as CMV and cause uveitis, which is also why CMV retinitis and IRU commonly co-exist.

Since IRU is closely related to CMV retinitis, the diagnosis of CMV retinitis should be mentioned. The diagnosis of CMV retinitis is simple, as the clinical features of CMV retinitis are easily recognized. An experienced ophthalmologist diagnoses CMV retinitis through indirect ophthalmoscopy by observing the characteristic patterns of CMV retinitis: (1) the “pizza” or “cottage cheese with ketchup retinopathy” presentation, which is characterized by centripetal necrotic retinal areas with associated hemorrhage. This is the most common presentation of CMV retinitis; (2) variable small dot-like lesions; and (3) retinal vasculitis with perivascular sheathing [69,70]. CMV retinitis causes full-thickness retinal necrosis, which leaves an atrophic and gliotic scar [71]. Compared with other opportunistic pathogens that involve the retina and choroid, CMV retinitis is more likely to be accompanied by minimal vitritis and iritis, following massive retinal necrosis, and fine, non-granulomatous, karetic precipitates on the corneal epithelium [72]. Early diagnosis is an effective way to reduce the incidence of blindness caused by CMV retinitis and the risk of IRU. However, in many HIV high-burden countries, a shortage of ophthalmologists is a prevailing problem. A study from Thailand showed that remote CMV retinitis diagnosis by trained nonexpert clinicians is satisfactory, and this might solve the ophthalmologist shortage [73].

Recently, few studies have focused on biomarkers for the diagnosis of IRU. Currently, the risk factors most associated with IRU are HIV viral load and CD4+ T cell count [74]. Duraikkannu et al. found that the expression of miRNA-192 in patients with IRU was significantly decreased, which might help us identify the status of PLWH [75]. Additionally, three metabolites were identified as the most specific indices to distinguish IRIS before ART: (i) oxidized cysteinyl-glycine (Cys-Gly Oxidized), (ii) 1-myristoyl-2-palmitoyl GPC (14:0/16:0), and (iii) the sulfate of piperine metabolite (C_18_H_21_NO_3_). However, after 1 month of ART, quinolinate, gluconate, and serine were the indices driving the distinction [76]. In addition, plasma tumor necrosis factor-α and mucin domain 3 levels were significantly increased after ART in HIV-infected patients with IRIS [77]. 

Typically, ART is initiated as soon as HIV is diagnosed. However, a study in 2005 demonstrated that delayed ART reduced the incidence and severity of CMV-associated IRU [78]. Controlling CMV retinitis before starting ART can significantly reduce the occurrence and severity of IRU [79]. Therefore, continuing anti-CMV treatment to minimize lesions until the immune system is strong enough to control retinitis is necessary [80]. Most experts recommend starting ART within one to two weeks after anti-CMV treatment [71]; however, further research is required to support this recommendation. Once an IRU is present, most clinicians recommend local or systemic steroid treatment based on the position and degree of ocular inflammation. However, with the application of steroids, TBU should be monitored because steroid use is an important risk factor for the occurrence of TBU [81,82].

### 3.4. Drug-Related Uveitis

Only 0.5% of uveitis cases are caused by drugs, [83] and related reports are limited. HIV-related uveitis generally presents as posterior segment disease. However, drug-induced uveitis in patients with HIV is mostly anterior uveitis. 

Most drug-induced uveitis cases are caused by cidofovir and rifabutin, although the use of these two drugs has greatly reduced in the ART era. Cidofovir is effective for treating CMV retinitis and is widely used in patients with HIV. However, there are reports on the occurrence of uveitis after using cidofovir. Anterior uveitis occurred in 26% of patients who used cidofovir [84]. Evidence supporting cidofovir-induced uveitis includes dose-related uveitis manifestation and probenecid, a drug against cidofovir, which is effective for cidofovir-induced uveitis [85,86]. Reduced intraocular pressure (IOP) can be observed in the development of cidofovir-induced uveitis. Thus, the administration of cidofovir should be assessed in patients with HIV with a low IOP. Once cidofovir-induced uveitis is diagnosed, steroids and cycloplegic/mydriatic agents can be administered as treatment [87]. Cidofovir-induced uveitis is more likely to occur in the eye with previous CMV disease [88], indicating the development of cidofovir-induced uveitis is related to immune recovery. The use of cidofovir could stimulate the immune system and thus increase the number of CD4+ T cells, causing ocular inflammation and leading to uveitis [89]. The stage of cidofovir-induced uveitis emergence is close to that of IRU; thus, cidofovir-induced uveitis is likely to be overlooked or misdiagnosed. However, the clinical manifestation of cidofovir-induced uveitis is quite different from that of IRU. Compared with cidofovir-induced uveitis, anterior or intermediate uveitis is more likely to be present in IRU, while posterior synechiae or granulomatous keratic precipitates are more common in cidofovir-induced uveitis. 

Rifabutin is commonly administered to treat *Mycobacterium avium* intracellular complex infection in patients with HIV whose CD4+ T cell count is <100 cells/µL. Recently, Nair et al. reported the occurrence of uveitis following rifabutin treatment as a complication of IRIS in patients with HIV [90]. The underlying mechanism of rifabutin-induced uveitis is related to drug toxicity and immune responses. Drug toxicity was directly proven by dose-dependent manifestation and recovery after adjusting the rifabutin dosage. The occurrence of uveitis after rifabutin administration is highly associated with combination treatment with clarithromycin [91,92], which is a CYP3A inhibitor that increases serum levels of rifabutin [93]. Therefore, a daily dose of <150 mg is administered when a protease inhibitor is combined with rifabutin. Rifabutin-induced uveitis is typically treated with corticosteroids and mydriatics with satisfactory outcomes.

## 4. Occupational Exposure to HIV in Ophthalmology

As the number of PLWH increases, healthcare workers, especially those in developing countries, are frequently at risk of exposure to the patients’ blood and body fluids. In general, the risk of HIV transmission from patients to healthcare workers is 0.3% [94]. The risk of HIV transmission through needlestick injury is approximately 0.3% [95], and the risk through mucous membrane exposure is 0.1%. Exposure to a large amount of blood from patients with late AIDS increases the risk of transmission, as exposure to blood is more dangerous than exposure to other body fluids, and serum viral load is an important risk factor [96,97]. More than 20% of patients living in parts of Southern Africa who needed surgical procedures were HIV positive, and surgeons have an average of three HIV exposures per person, per year [98]. In 2005, the WHO reported that approximately 3 million percutaneous exposures to healthcare workers occur annually, and the most common exposure is through accidental puncture by a needle or other sharp devices [99]. Furthermore, according to research from India, only half of the occupational exposure was reported [100]. Therefore, the risk of HIV exposure to healthcare workers might be largely underestimated, and healthcare workers, especially surgeons, are considered a high-risk population of HIV infection.

Although ophthalmologists are not generally considered to be at high risk of HIV infection, they should be aware of HIV protection. Ophthalmologists can be exposed to HIV through mucous membrane contact during medical treatment, drainage of aqueous/vitreous humor, injection procedure, and surgery. Therefore, it is necessary for ophthalmologists to improve their awareness of occupational exposure to HIV. Further research is needed on the extent of ophthalmologists’ exposure to HIV and the prevention measures.

Most clinicians from developing countries are reported to lack knowledge about occupational exposure and protection measures against HIV [101]. All healthcare workers, including ophthalmologists, should be trained on standard prevention measures for occupational exposure. Increased awareness will reduce HIV-related stigma and discrimination among healthcare workers. Furthermore, health and safety guidelines should be placed in the working station or easily visible places for quick reference. Healthcare workers should be provided with personal protective equipment (PPE), such as boots, aprons, masks, and glasses, to prevent exposure to body fluids during an operation. It is appalling that healthcare workers in public hospitals in developing countries reuse PPE due to resource constraints, and this problem was exacerbated by the coronavirus disease pandemic [102]. For healthcare workers, proper handling of infectious materials and adhering to the correct waste disposal protocol is a common but easily overlooked problem [102]. Almost half of the healthcare workers will not report an incident of exposure to HIV [103], which negates the use of post-exposure prophylaxis (PEP). Nevertheless, ART remains a priority for protecting healthcare workers from HIV infection, and one should take PEP within 72 h after HIV exposure. Overall, a standard occupation protocol should be established, and healthcare workers should be encouraged to adhere to it. 

## 5. Conclusions

As ART becomes widespread and the survival of patients with HIV increases, quality of life has become a new issue of concern. The causes of uveitis in patients with HIV could be complex. Hence, raising awareness of uveitis and its clinical manifestation is important for both patients with HIV and infectious disease physicians. When patients with HIV present with uveitis-related changes, a complete systematic assessment should be started, including the duration and treatment of HIV, CD4+ T cell count, HIV viral loads, and serological tests. Early diagnosis and treatment can help achieve optimal outcomes and a higher quality of life for patients with HIV. Although occupational exposure to HIV is very rare, ophthalmologists should still be familiar with the precautions and management of occupational exposure to HIV. 

## Data Availability

All data related to this study are presented and published here.

## References

[1] HIV/AIDS JUNPo. 2020 Global AIDS Update Seizing the Moment-Tackling Entrenched Inequalities to End Epidemics. https://www.unaids.org/en/resources/documents/2020/global-aids-report.

[2] Murray C.J., Ortblad K.F., Guinovart C., Lim S.S., Wolock T.M., Roberts D.A., Dansereau E.A., Graetz N., Barber R.M., Brown J.C. (2014). Global, regional, and national incidence and mortality for HIV, tuberculosis, and malaria during 1990–2013: A systematic analysis for the Global Burden of Disease Study 2013. Lancet.

[3] Delpech V. (2022). The HIV epidemic: Global and United Kingdom trends. Medicine.

[4] Arora R., Sandhu N., Dokania P., Subramanian A. (2021). Ocular Manifestations in Patients of HIV(Human Immunodeficiency Virus) Infection on Combined Anti-Retroviral Therapy (CART). Ocul. Immunol. Inflamm..

[5] Hodge W.G., Seiff S.R., Margolis T.P. (1998). Ocular opportunistic infection incidences among patients who are HIV positive compared to patients who are HIV negative. Ophthalmology.

[6] de-la-Torre A. (2018). Virus-Induced Anterior Uveitis (VIAU) in Immunocompromised Patients. Ocul. Immunol. Inflamm..

[7] Becker K.N., Becker N.M. (2014). Ocular manifestations seen in HIV. Dis. Mon..

[8] Saini N., Hasija S., Kaur P., Kaur M., Pathania V., Singh A. (2019). Study of prevalence of ocular manifestations in HIV positive patients. Nepal. J. Ophthalmol..

[9] Hoover D.R., Peng Y., Saah A., Semba R., Detels R.R., Rinaldo C.R., Phair J.P. (1996). Occurrence of cytomegalovirus retinitis after human immunodeficiency virus immunosuppression. Arch. Ophthalmol..

[10] Sugar E.A., Jabs D.A., Ahuja A., Thorne J.E., Danis R.P., Meinert C.L. (2012). Incidence of cytomegalovirus retinitis in the era of highly active antiretroviral therapy. Am. J. Ophthalmol..

[11] Holland G.N. (2008). AIDS and ophthalmology: The first quarter century. Am. J. Ophthalmol..

[12] Sudharshan S., Nair N., Curi A., Banker A., Kempen J.H. (2020). Human immunodeficiency virus and intraocular inflammation in the era of highly active anti retroviral therapy—An update. Indian J. Ophthalmol..

[13] Sadik M.T., Aksu Ceylan N., Cebeci Z., Kir N., Oray M., Tugal-Tutkun I. (2021). Patterns of cytomegalovirus retinitis at a tertiary referral center in Turkey. Int. Ophthalmol..

[14] Saadouli D., Ammari L., Ben Mansour K., Yahyaoui Y., Aissa S., Mohamed Ali E.A., Yahyaoui S., Tiouri H. (2021). Ocular manifestations of people living with HIV in Tunisia. South. Afr. J. HIV Med..

[15] Rothova A., Hajjaj A., de Hoog J., Thiadens A., Dalm V. (2019). Uveitis causes according to immune status of patients. Acta Ophthalmol..

[16] Persidsky Y., Poluektova L. (2006). Immune privilege and HIV-1 persistence in the CNS. Immunol. Rev..

[17] Rothova A., Schneider M., de Groot-Mijnes J.D. (2008). Human immunodeficiency virus-induced uveitis: Intraocular and plasma human immunodeficiency virus-1 RNA loads. Ophthalmology.

[18] Nussenblatt R.B., Lane H.C. (1998). Human immunodeficiency virus disease: Changing patterns of intraocular inflammation. Am. J. Ophthalmol..

[19] Cantrill H.L., Henry K., Jackson B., Erice A., Ussery F.M., Balfour H.H. (1988). Recovery of human immunodeficiency virus from ocular tissues in patients with acquired immune deficiency syndrome. Ophthalmology.

[20] Pham V.T., Wen L., McCluskey P., Madigan M.C., Penfold P.L. (2005). Human retinal microglia express candidate receptors for HIV-1 infection. Br. J. Ophthalmol..

[21] Rummelt V., Rummelt C., Jahn G., Wenkel H., Sinzger C., Mayer U.M., Naumann G.O. (1994). Triple retinal infection with human immunodeficiency virus type 1, cytomegalovirus, and herpes simplex virus type 1. Light and electron microscopy, immunohistochemistry, and in situ hybridization. Ophthalmology.

[22] Pathanapitoon K., Riemens A., Kongyai N., Sirirungsi W., Leechanachai P., Ausayakhun S., Kalinina Ayuso V., Kunavisarut P., de Groot-Mijnes J.D., Rothova A. (2011). Intraocular and plasma HIV-1 RNA loads and HIV uveitis. AIDS.

[23] Kunavisarut P., Sirirungsi W., Pathanapitoon K., Rothova A. (2012). Clinical manifestations of human immunodeficiency virus-induced uveitis. Ophthalmology.

[24] Che X., He F., Deng Y., Xu S., Fan X., Gu P., Wang Z. (2014). HIV-1 Tat-mediated apoptosis in human blood-retinal barrier-associated cells. PLoS ONE.

[25] Krishnan G., Chatterjee N. (2015). Differential immune mechanism to HIV-1 Tat variants and its regulation by AEA [corrected]. Sci. Rep..

[26] Oliver S.E., Aubin M., Atwell L., Matthias J., Cope A., Mobley V., Goode A., Minnerly S., Stoltey J., Bauer H.M. (2016). Ocular Syphilis—Eight Jurisdictions, United States, 2014–2015. MMWR Morb. Mortal. Wkly. Rep..

[27] Hughes E.H., Guzowski M., Simunovic M.P., Hunyor A.P., McCluskey P. (2010). Syphilitic retinitis and uveitis in HIV-positive adults. Clin. Exp. Ophthalmol..

[28] Traeger M.W., Cornelisse V.J., Asselin J., Price B., Roth N.J., Willcox J., Tee B.K., Fairley C.K., Chang C.C., Armishaw J. (2019). Association of HIV Preexposure Prophylaxis With Incidence of Sexually Transmitted Infections Among Individuals at High Risk of HIV Infection. JAMA.

[29] Chesson H.W., Heffelfinger J.D., Voigt R.F., Collins D. (2005). Estimates of primary and secondary syphilis rates in persons with HIV in the United States, 2002. Sex. Transm. Dis..

[30] Rose-Nussbaumer J., Goldstein D.A., Thorne J.E., Arantes T.E., Acharya N.R., Shakoor A., Jeng B.H., Yeh S., Rahman H., Vemulakonda G.A. (2014). Uveitis in human immunodeficiency virus-infected persons with CD4+ T-lymphocyte count over 200 cells/mL. Clin. Exp. Ophthalmol..

[31] Mathew D., Smit D. (2021). Clinical and laboratory characteristics of ocular syphilis and neurosyphilis among individuals with and without HIV infection. Br. J. Ophthalmol..

[32] Bollemeijer J.G., Wieringa W.G., Missotten T.O., Meenken I., ten Dam-van Loon N.H., Rothova A., Los L.I. (2016). Clinical Manifestations and Outcome of Syphilitic Uveitis. Invest. Ophthalmol. Vis. Sci..

[33] Seña A.C., White B.L., Sparling P.F. (2010). Novel Treponema pallidum serologic tests: A paradigm shift in syphilis screening for the 21st century. Clin. Infect. Dis..

[34] Forrestel A.K., Kovarik C.L., Katz K.A. (2020). Sexually acquired syphilis: Laboratory diagnosis, management, and prevention. J. Am. Acad. Dermatol..

[35] Centers for Disease Control and Prevention (2008). Syphilis testing algorithms using treponemal tests for initial screening--four laboratories, New York City, 2005-2006. MMWR Morb Mortal Wkly. Rep..

[36] Centers for Disease Control and Prevention (2011). Discordant results from reverse sequence syphilis screening--five laboratories, United States, 2006-2010. MMWR Morb. Mortal. Wkly. Rep..

[37] Workowski K.A., Bachmann L.H., Chan P.A., Johnston C.M., Muzny C.A., Park I., Reno H., Zenilman J.M., Bolan G.A. (2021). Sexually Transmitted Infections Treatment Guidelines, 2021. MMWR Recomm. Rep..

[38] Tyagi M., Kaza H., Pathengay A., Agrawal H., Behera S., Lodha D., Pappuru R.R., Basu S., Murthy S. (2020). Clinical manifestations and outcomes of ocular syphilis in Asian Indian population: Analysis of cases presenting to a tertiary referral center. Indian J. Ophthalmol..

[39] Oliver G.F., Stathis R.M., Furtado J.M., Arantes T.E., McCluskey P.J., Matthews J.M., International Ocular Syphilis Study G., Smith J.R. (2019). Current ophthalmology practice patterns for syphilitic uveitis. Br. J. Ophthalmol..

[40] Rasoldier V., Gueudry J., Chapuzet C., Bodaghi B., Muraine M., Tubiana R., Paris L., Pestel-Caron M., Caron F., Caumes E. (2021). Early symptomatic neurosyphilis and ocular syphilis: A comparative study between HIV-positive and HIV-negative patients. Infect. Dis. Now..

[41] Janier M., Unemo M., Dupin N., Tiplica G.S., Potočnik M., Patel R. (2021). 2020 European guideline on the management of syphilis. J. Eur. Acad. Dermatol. Venereol..

[42] Testi I., Ahmed S., Shah C., Agrawal R. (2020). Challenges in Treating Intraocular Inflammation in HIV Patients. Ocul. Immunol. Inflamm..

[43] Testi I., Mahajan S., Agrawal R., Agarwal A., Marchese A., Curi A., Khairallah M., Leo Y.S., Nguyen Q.D., Gupta V. (2020). Management of Intraocular Infections in HIV. Ocul. Immunol. Inflamm..

[44] Sudharshan S., Menia N.K., Selvamuthu P., Tyagi M., Kumarasamy N., Biswas J. (2020). Ocular syphilis in patients with human immunodeficiency virus/acquired immunodeficiency syndrome in the era of highly active antiretroviral therapy. Indian J. Ophthalmol..

[45] Hoogewoud F., Frumholtz L., Loubet P., Charlier C., Blanche P., Lebeaux D., Benhaddou N., Sedira N., Coutte L., Vanhaecke C. (2017). Prognostic Factors in Syphilitic Uveitis. Ophthalmology.

[46] Wu M.Y., Gong H.Z., Hu K.R., Zheng H.Y., Wan X., Li J. (2021). Effect of syphilis infection on HIV acquisition: A systematic review and meta-analysis. Sex. Transm. Infect..

[47] De Cock K.M., Jaffe H.W., Curran J.W. (2012). The evolving epidemiology of HIV/AIDS. Aids.

[48] Mesfin Y.M., Hailemariam D., Biadgilign S., Kibret K.T. (2014). Association between HIV/AIDS and multi-drug resistance tuberculosis: A systematic review and meta-analysis. PLoS ONE.

[49] Bell L.C.K., Noursadeghi M. (2018). Pathogenesis of HIV-1 and Mycobacterium tuberculosis co-infection. Nat. Rev. Microbiol..

[50] Getahun H., Gunneberg C., Granich R., Nunn P. (2010). HIV infection-associated tuberculosis: The epidemiology and the response. Clin. Infect. Dis..

[51] Gupta R.K., Lucas S.B., Fielding K.L., Lawn S.D. (2015). Prevalence of tuberculosis in post-mortem studies of HIV-infected adults and children in resource-limited settings: A systematic review and meta-analysis. Aids.

[52] Agrawal R., Gunasekeran D.V., Grant R., Agarwal A., Kon O.M., Nguyen Q.D., Pavesio C., Gupta V. (2017). Clinical Features and Outcomes of Patients With Tubercular Uveitis Treated With Antitubercular Therapy in the Collaborative Ocular Tuberculosis Study (COTS)-1. JAMA Ophthalmol..

[53] La Distia Nora R., Sitompul R., Bakker M., Susiyanti M., Edwar L., Sjamsoe S., Singh G., van Hagen M.P., Rothova A. (2018). Tuberculosis and other causes of uveitis in Indonesia. Eye.

[54] Lin C.J., Hsia N.Y., Hwang D.K., Hwang Y.S., Chang Y.C., Lee Y.C., Hsu Y.R., Yeh P.T., Lin C.P., Chen H.F. (2022). Clinical Manifestations and Outcomes of Tubercular Uveitis in Taiwan-A Ten-Year Multicenter Retrospective Study. Medicina.

[55] Markowitz N., Hansen N.I., Hopewell P.C., Glassroth J., Kvale P.A., Mangura B.T., Wilcosky T.C., Wallace J.M., Rosen M.J., Reichman L.B. (1997). Incidence of tuberculosis in the United States among HIV-infected persons. The Pulmonary Complications of HIV Infection Study Group. Ann. Intern. Med..

[56] Miyahara R., Piyaworawong S., Prachamat P., Wongyai J., Bupachat S., Yamada N., Summanapan S., Yanai H., Mahasirimongkol S. (2020). High tuberculosis burden among HIV-infected populations in Thailand due to a low-sensitivity tuberculin skin test. J. Infect. Public Health.

[57] Moon H.W., Hur M. (2013). Interferon-gamma release assays for the diagnosis of latent tuberculosis infection: An updated review. Ann. Clin. Lab. Sci.

[58] Mehta S., Peters R.P., Smit D.P., Gupta V. (2020). Ocular Tuberculosis in HIV-infected Individuals. Ocul. Immunol. Inflamm..

[59] Smit D.P., Esterhuizen T.M., Meyer D. (2018). The Role of QuantiFERON(^®^)-TB Gold and Tuberculin Skin Test as Diagnostic Tests for Intraocular Tuberculosis in HIV-Positive and HIV-Negative Patients in South Africa. Ocul. Immunol. Inflamm..

[60] Tomkins-Netzer O., Leong B.C.S., Zhang X., Lightman S., McCluskey P.J. (2018). Effect of Antituberculous Therapy on Uveitis Associated With Latent Tuberculosis. Am. J. Ophthalmol..

[61] Agrawal R., Gunasekeran D.V., Raje D., Agarwal A., Nguyen Q.D., Kon O.M., Pavesio C., Gupta V. (2018). Global Variations and Challenges With Tubercular Uveitis in the Collaborative Ocular Tuberculosis Study. Invest. Ophthalmol. Vis. Sci..

[62] Agrawal R., Testi I., Bodaghi B., Barisani-Asenbauer T., McCluskey P., Agarwal A., Kempen J.H., Gupta A., Smith J.R., de Smet M.D. (2021). Collaborative Ocular Tuberculosis Study Consensus Guidelines on the Management of Tubercular Uveitis-Report 2: Guidelines for Initiating Antitubercular Therapy in Anterior Uveitis, Intermediate Uveitis, Panuveitis, and Retinal Vasculitis. Ophthalmology.

[63] Laovirojjanakul W., Thanathanee O. (2018). Opportunistic ocular infections in the setting of HIV. Curr. Opin. Ophthalmol..

[64] Schaftenaar E., Meenken C., Baarsma G.S., Verjans G.M., Peters R.P. (2014). Good visual outcome of tuberculous chorioretinitis after ART initiation in a HIV-infected patient. Int. Ophthalmol..

[65] Sudharshan S., Kaleemunnisha S., Banu A.A., Shrikrishna S., George A.E., Babu B.R., Devaleenal B., Kumarasamy N., Biswas J. (2013). Ocular lesions in 1,000 consecutive HIV-positive patients in India: A long-term study. J. Ophthalmic. Inflamm. Infect..

[66] Ratnam I., Chiu C., Kandala N.B., Easterbrook P.J. (2006). Incidence and risk factors for immune reconstitution inflammatory syndrome in an ethnically diverse HIV type 1-infected cohort. Clin. Infect. Dis..

[67] Yeo T.H., Yeo T.K., Wong E.P., Agrawal R., Teoh S.C. (2016). Immune recovery uveitis in HIV patients with cytomegalovirus retinitis in the era of HAART therapy-a 5-year study from Singapore. J. Ophthalmic. Inflamm. Infect..

[68] Schrier R.D., Song M.K., Smith I.L., Karavellas M.P., Bartsch D.U., Torriani F.J., Garcia C.R., Freeman W.R. (2006). Intraocular viral and immune pathogenesis of immune recovery uveitis in patients with healed cytomegalovirus retinitis. Retina.

[69] Port A.D., Orlin A., Kiss S., Patel S., D’Amico D.J., Gupta M.P. (2017). Cytomegalovirus Retinitis: A Review. J. Ocul. Pharmacol. Ther..

[70] Wons J., Kempen J., Garweg J.G. (2020). HIV-induced Retinitis. Ocul. Immunol. Inflamm..

[71] Jabs D.A. (2011). Cytomegalovirus retinitis and the acquired immunodeficiency syndrome--bench to bedside: LXVII Edward Jackson Memorial Lecture. Am. J. Ophthalmol..

[72] Zegans M.E., Walton R.C., Holland G.N., O’Donnell J.J., Jacobson M.A., Margolis T.P. (1998). Transient vitreous inflammatory reactions associated with combination antiretroviral therapy in patients with AIDS and cytomegalovirus retinitis. Am. J. Ophthalmol..

[73] Yen M., Ausayakhun S., Chen J., Ausayakhun S., Jirawison C., Heiden D., Holland G.N., Margolis T.P., Keenan J.D. (2014). Telemedicine diagnosis of cytomegalovirus retinitis by nonophthalmologists. JAMA Ophthalmol..

[74] Müller M., Wandel S., Colebunders R., Attia S., Furrer H., Egger M. (2010). Immune reconstitution inflammatory syndrome in patients starting antiretroviral therapy for HIV infection: A systematic review and meta-analysis. Lancet Infect. Dis..

[75] Duraikkannu D., Akbar A.B., Sudharshan S., Poongulali S., Kumarasamy N., Jayavelu T., Chatterjee N. (2022). Differential Expression of miRNA-192 is a Potential Biomarker for HIV Associated Immune Recovery Uveitis. Ocul. Immunol. Inflamm..

[76] Pei L., Fukutani K.F., Tibúrcio R., Rupert A., Dahlstrom E.W., Galindo F., Laidlaw E., Lisco A., Manion M., Andrade B.B. (2021). Plasma Metabolomics Reveals Dysregulated Metabolic Signatures in HIV-Associated Immune Reconstitution Inflammatory Syndrome. Front. Immunol..

[77] Ramon-Luing L.A., Ocaña-Guzman R., Téllez-Navarrete N.A., Preciado-García M., Romero-Rodríguez D.P., Espinosa E., Reyes-Terán G., Chavez-Galan L. (2021). High Levels of TNF-α and TIM-3 as a Biomarker of Immune Reconstitution Inflammatory Syndrome in People with HIV Infection. Life.

[78] Ortega-Larrocea G., Espinosa E., Reyes-Terán G. (2005). Lower incidence and severity of cytomegalovirus-associated immune recovery uveitis in HIV-infected patients with delayed highly active antiretroviral therapy. Aids.

[79] Kuppermann B.D., Holland G.N. (2000). Immune recovery uveitis. Am. J. Ophthalmol..

[80] National Institutes of Health, Centers for Disease Control and Prevention, HIV Medicine Association, Infectious Diseases Society of America Panel on Guidelines for the Prevention and Treatment of Opportunistic Infections in Adults and Adolescents with HIV. Guidelines for the Prevention and Treatment of Opportunistic Infections in Adults and Adolescents with HIV. https://clinicalinfo.hiv.gov/en/guidelines/hiv-clinical-guidelines-adult-and-adolescent-opportunistic-infections/whats-new.

[81] Agarwal A., Handa S., Aggarwal K., Sharma M., Singh R., Sharma A., Agrawal R., Sharma K., Gupta V. (2018). The Role of Dexamethasone Implant in the Management of Tubercular Uveitis. Ocul. Immunol. Inflamm..

[82] Jain L., Panda K.G., Basu S. (2018). Clinical Outcomes of Adjunctive Sustained-Release Intravitreal Dexamethasone Implants in Tuberculosis-Associated Multifocal Serpigenoid Choroiditis. Ocul. Immunol. Inflamm..

[83] Fraunfelder F.W., Rosenbaum J.T. (1997). Drug-induced uveitis. Incidence, prevention and treatment. Drug Saf..

[84] Davis J.L., Taskintuna I., Freeman W.R., Weinberg D.V., Feuer W.J., Leonard R.E. (1997). Iritis and hypotony after treatment with intravenous cidofovir for cytomegalovirus retinitis. Arch. Ophthalmol..

[85] Kirsch L.S., Arevalo J.F., Chavez de la Paz E., Munguia D., de Clercq E., Freeman W.R. (1995). Intravitreal cidofovir (HPMPC) treatment of cytomegalovirus retinitis in patients with acquired immune deficiency syndrome. Ophthalmology.

[86] Chavez-de la Paz E., Arevalo J.F., Kirsch L.S., Munguia D., Rahhal F.M., De Clercq E., Freeman W.R. (1997). Anterior nongranulomatous uveitis after intravitreal HPMPC (cidofovir) for the treatment of cytomegalovirus retinitis. Analysis and prevention. Ophthalmology.

[87] Testi I., Agarwal A., Agrawal R., Mahajan S., Marchese A., Miserocchi E., Gupta V. (2020). Drug-induced Uveitis in HIV Patients with Ocular Opportunistic Infections. Ocul. Immunol. Inflamm..

[88] Bainbridge J.W., Raina J., Shah S.M., Ainsworth J., Pinching A.J. (1999). Ocular complications of intravenous cidofovir for cytomegalovirus retinitis in patients with AIDS. Eye.

[89] Ambati J., Wynne K.B., Angerame M.C., Robinson M.R. (1999). Anterior uveitis associated with intravenous cidofovir use in patients with cytomegalovirus retinitis. Br. J. Ophthalmol..

[90] Nair N., Sudharshan S., Koladiya N.A., Biswas J. (2022). Rifabutin induced hypopyon uveitis mimicking endophthalmitis as a manifestation of IRU in patients with HIV. Indian J. Pharmacol..

[91] Shafran S.D., Deschênes J., Miller M., Phillips P., Toma E. (1994). Uveitis and pseudojaundice during a regimen of clarithromycin, rifabutin, and ethambutol. MAC Study Group of the Canadian HIV Trials Network. N. Engl. J. Med..

[92] Kelleher P., Helbert M., Sweeney J., Anderson J., Parkin J., Pinching A. (1996). Uveitis associated with rifabutin and macrolide therapy for Mycobacterium avium intracellulare infection in AIDS patients. Genitourin. Med..

[93] Agarwal M., Dutta Majumder P., Babu K., Konana V.K., Goyal M., Touhami S., Stanescu-Segall D., Bodaghi B. (2020). Drug-induced uveitis: A review. Indian J. Ophthalmol..

[94] Saltzman D.J., Williams R.A., Gelfand D.V., Wilson S.E. (2005). The surgeon and AIDS: Twenty years later. Arch. Surg..

[95] Asanati K., Majeed A., Shemtob L., Cresswell F. (2022). Healthcare workers potentially exposed to HIV: An update. J. R. Soc. Med..

[96] Cardo D.M., Culver D.H., Ciesielski C.A., Srivastava P.U., Marcus R., Abiteboul D., Heptonstall J., Ippolito G., Lot F., McKibben P.S. (1997). A case-control study of HIV seroconversion in health care workers after percutaneous exposure. Centers for Disease Control and Prevention Needlestick Surveillance Group. N. Engl. J. Med..

[97] Kuhar D.T.H., David K., Struble K.A., Heneine W., Thomas V., Cheever L.W., Gomaa A., Panlilio A.L., USPHS Working Group on Occupational Postexposure Prophylaxis, National Center for Emerging and Zoonotic Infectious Diseases (U.S.) Updated U.S. Public Health Service Guidelines for the Management of Occupational Exposures to HIV and Recommendations for Postexposure Prophylaxis. https://stacks.cdc.gov/view/cdc/20711.

[98] Kingham T.P., Kamara T.B., Daoh K.S., Kabbia S., Kushner A.L. (2009). Universal precautions and surgery in Sierra Leone: The unprotected workforce. World J. Surg..

[99] Prüss-Ustün A., Rapiti E., Hutin Y. (2005). Estimation of the global burden of disease attributable to contaminated sharps injuries among health-care workers. Am. J. Ind. Med..

[100] Doebbeling B.N., Vaughn T.E., McCoy K.D., Beekmann S.E., Woolson R.F., Ferguson K.J., Torner J.C. (2003). Percutaneous injury, blood exposure, and adherence to standard precautions: Are hospital-based health care providers still at risk?. Clin. Infect. Dis..

[101] Wu Q., Xue X.F., Shah D., Zhao J., Hwang L.Y., Zhuang G. (2016). Knowledge, Attitude, and Practices Regarding Occupational HIV Exposure and Protection among Health Care Workers in China: Census Survey in a Rural Area. J. Int. Assoc. Provid. AIDS Care.

[102] Mashoto K.O., Mubyazi G.M., Makundi E., Mohamed H., Malebo H.M. (2013). Estimated risk of HIV acquisition and practice for preventing occupational exposure: A study of healthcare workers at Tumbi and Dodoma Hospitals, Tanzania. BMC Health Serv. Res..

[103] Kumakech E., Achora S., Berggren V., Bajunirwe F. (2011). Occupational exposure to HIV: A conflict situation for health workers. Int. Nurs. Rev..

